# Effects of formative-summative assessment weight configurations on evaluation validity and discriminatory power in landscape architecture planning and design courses

**DOI:** 10.1038/s41598-026-50554-7

**Published:** 2026-04-27

**Authors:** Dong Dong, Mingxin Chen, Shaojie Zhang, Yongxin Chen, Fengquan Ji

**Affiliations:** 1 School of Architecture and Urban Planning, Anhui Jianzhu University, Hefei, 230601 Anhui China; 2 College of Horticulture and Forestry Sciences, Huazhong Agricultural University, Wuhan, 430070 Hubei China; 3 Anhui Institute of Territorial Spatial Planning and Ecology, Hefei, 230601 Anhui China

**Keywords:** Accreditation assessment, Landscape architecture planning and design, Formative assessment, Summative assessment, Assessment weighting, Validity, Discrimination, Engineering, Mathematics and computing, Psychology, Psychology

## Abstract

This study investigates how different formative–summative assessment weight configurations affect evaluation validity and discriminatory power in a landscape architecture planning and design course, in the context of contemporary undergraduate education evaluation reforms in China. A randomized controlled experiment with 90 landscape architecture students at a provincial university in eastern China implemented three weight allocation schemes: 30%:70%, 50%:50%, and 70%:30% (formative: summative). Validity analysis, discrimination analysis, and curve fitting were used to examine the effects of weight configuration on assessment quality. The findings indicate that (1) weight configuration has a pronounced nonlinear effect on evaluation validity: the balanced configuration (50%:50%) yielded higher validity (r = 0.845) than both the traditional (30%:70%, r = 0.723) and process-oriented configurations (70%:30%, r = 0.776), with validity approaching its optimum near a formative weight of approximately 53%; (2) the effect of weight configuration on discriminatory power exhibits diminishing marginal returns: the process-oriented configuration produced the highest discrimination index (D = 0.435), followed by the balanced configuration (D = 0.412), both substantially exceeding the traditional configuration (D = 0.325)—when validity and discriminatory power are considered jointly, a formative weight of approximately 55% yielded stronger overall performance; and (3) different competency dimensions respond differentially to assessment approaches—site analysis and design thinking showed higher validity under formative assessment, whereas spatial composition and communication skills were more effectively captured by summative assessment. This study provides empirical evidence and an adaptable methodological framework for evidence-based refinement of assessment systems in design courses.

**Supplementary Information:**

The online version contains supplementary material available at 10.1038/s41598-026-50554-7.

## Introduction

The current round of undergraduate education evaluation is a cornerstone of China’s higher education quality assurance framework, reshaping the trajectory of university educational reform^1^. Initiated by the Ministry of Education in 2021, this paradigm embraces three foundational principles: student-centered, outcome-oriented, and continuous improvement. This shift in emphasis—from “what to teach” toward “what students learn” and “how effectively they learn”^2^—resonates with outcome-based education principles underpinning quality assurance movements worldwide^3^. Within this framework, the rigor of course assessment systems directly affects judgments of educational quality^3^, and for landscape architecture—a discipline integrating artistic creativity with technical precision—evaluating professional competency remains a pressing challenge^4^.

How to allocate weights between formative and summative assessment in design courses remains a contested issue^5,6^. Traditional evaluation frameworks predominantly rely on summative assessment. Although this approach offers simplicity, it suffers from inherent limitations: subjective bias, narrow evaluative scope, and an inability to capture competency development processes^7,8^. As educational theory has advanced, formative assessment has gained prominence for its capacity to document learning trajectories and capture competency formation^9,10^, and contemporary design education increasingly adopts integrated formative–summative frameworks^11^. Yet determining how to distribute weights between these approaches remains inadequately supported by empirical evidence.

Existing research on this issue is characterized by abundant theoretical discussion but limited empirical validation, and by numerous experiential recommendations unsupported by quantitative evidence^12^. For example, some scholars recommend a 60:40 process-to-outcome configuration^13^ while others propose a 40:60 arrangement^14^, yet neither recommendation has been substantiated by quantitative evidence on assessment quality^15^. Moreover, existing studies generally overlook how weight configuration differentially affects the assessment of individual competency dimensions^16^. This empirical gap persists in part because assessment methods in design courses are deeply embedded in their respective studio traditions and accreditation systems. Across different educational systems worldwide, the tension between studio culture and standardized assessment varies considerably, and the boundaries between formative and summative assessment—along with implementation conventions—differ substantially^17,18^. Consequently, optimizing weight configuration cannot proceed through abstract deduction detached from specific educational contexts; it requires empirical investigation grounded in particular institutional conditions and disciplinary accreditation frameworks.

In educational measurement theory, validity and discriminatory power are the two core indicators of assessment quality^19–21^. Formative assessment documents and facilitates the process of competency formation, whereas summative assessment renders comprehensive judgments on learning outcomes at the conclusion of instruction^22^; the two are complementary rather than opposed, and only their integration can fully capture students’ professional competencies^23^. However, this framework addresses whether the two modes should be combined without answering a deeper question: whether different competency dimensions respond differentially to each mode, and what cognitive mechanisms underlie such responses. Research on design cognition indicates that design activity confronts ill-structured problems whose key cognitive operations—problem framing, strategy adjustment, and solution evaluation—are embedded in the act of designing itself, so that the final product cannot fully reflect the quality of these processes^24,25^. This implies that professional competencies differ fundamentally in their temporal observability^26^: competency dimensions relying on process-oriented cognition and those depending on integrative output may differ systematically in their compatibility with assessment modes, yet this hypothesis remains untested in design course assessment.

The “Landscape Architecture Planning and Design” course is a core professional course in landscape architecture whose competency structure informs the design of the assessment system. The course encompasses five core dimensions: site analysis capability, design thinking and innovation capability, spatial composition capability, technical application proficiency, and communication and presentation skills^27^. These dimensions are expressed differently across phases of the design process, and prior research suggests that certain competencies are better captured through formative assessment while others are more readily observed through summative assessment^23,28^. This heterogeneity makes the course well suited for testing the hypotheses outlined above^29^.

Against this background, the present study addresses three research questions: How do different formative–summative weight configurations affect evaluation validity and discriminatory power? Do different competency dimensions respond differentially to the two assessment modes, and if so, what patterns characterize these responses? When validity and discriminatory power are considered jointly, is there a favorable weight configuration interval? To address these questions, a randomized controlled experiment compared the assessment outcomes of three weight configurations (30%:70%, 50%:50%, and 70%:30%) in an authentic educational setting, using validity analysis, discrimination analysis, and curve fitting. The study aims to provide data-informed references for weight configuration in Chinese design courses while offering a replicable methodological framework adaptable to other design disciplines and educational contexts.

## Research methods

**Fig. 1:**
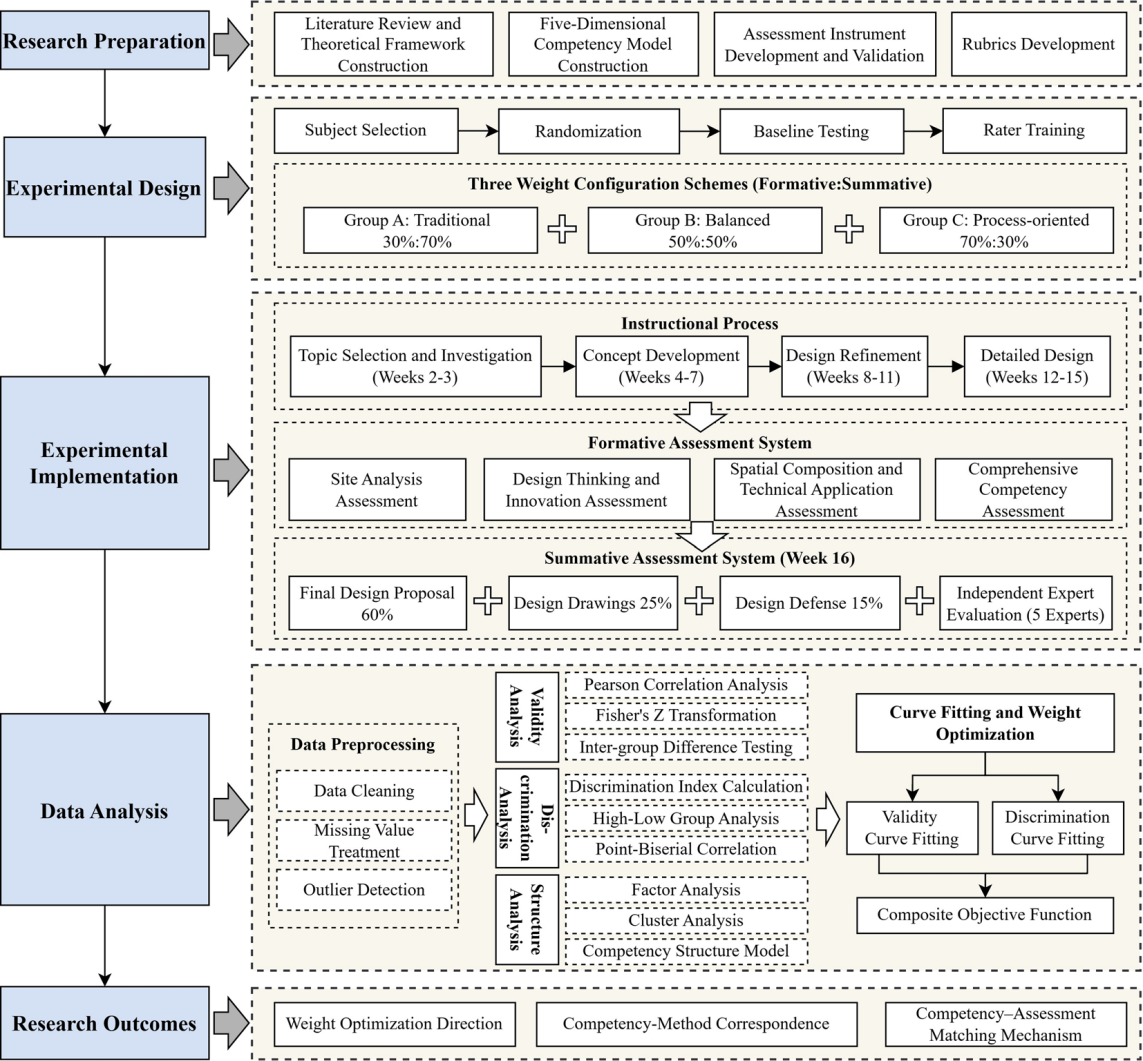
Research methodology framework.

A randomized controlled experimental design was employed to examine how different weight configurations affect evaluation quality in an authentic educational setting. The research proceeded through four phases—framework construction, tool development, experimental implementation, and model fitting—as illustrated in Fig. 1. The design randomly allocated 90 students to three groups, each assigned a different formative–summative weight configuration (30%:70%, 50%:50%, 70%:30%). While maintaining identical teaching content and evaluation standards, and balancing internal validity (rigorous experimental controls) with external validity (authentic settings), the study compared evaluation effectiveness across configurations through validity analysis and discrimination analysis.

### Participants and group assignment

Participants were undergraduate students from the 2021 and 2022 cohorts of the Landscape Architecture Program at Anhui Jianzhu University, with the “Landscape Architecture Planning and Design” course as the experimental vehicle. This core course carries 4 credits over 96 instructional hours (16 h of theory, 80 h of practice), typically delivered in the fourth semester. Course content spans site investigation, concept development, design refinement, detailed design, and presentation; its project-based, applied character makes it well suited for examining core professional competencies.

Ninety students were randomly assigned to three equal groups of 30. Sample size was determined by a priori power analysis using G*Power 3.1^30^: with a medium effect size (f = 0.35), α = 0.05, and 1 − β = 0.85, the minimum required sample for one-way ANOVA was 84, so the 90-participant sample provided adequate statistical power. To ensure group equivalence, baseline assessments of drawing proficiency, spatial cognitive ability, and professional foundational knowledge were conducted prior to randomization. One-way ANOVA confirmed no significant intergroup differences on any of these indicators (*p* > 0.05), validating group equivalence (see Table 2 for details).

The three experimental conditions were: Group A, traditional weighting (30% formative, 70% summative); Group B, balanced weighting (50% formative, 50% summative); and Group C, process-oriented weighting (70% formative, 30% summative).

This educational research was approved by the Science and Technology Ethics Committee of Anhui Jianzhu University (Approval No. 2023006, approved on 31 July 2023). The study was conducted in accordance with the Declaration of Helsinki and institutional guidelines for minimal-risk educational research. At the beginning of the course, the research team explained the research purpose and procedures to all participants and obtained verbal informed consent, documented through signed course attendance records. All participants were adults (aged 18–22 years). All data were completely anonymized, with no personally identifiable information retained in the dataset. To maintain experimental integrity, students were not informed of their specific group assignments during the study period, with full debriefing provided upon course completion.

### Experimental design and implementation

#### Experimental framework

The study used a naturalistic experimental design within an authentic educational setting over one complete semester (16 weeks). The experimental content followed the established curriculum for “Landscape Architecture Planning and Design,” encompassing five phases: topic selection and investigation, concept development, design refinement, detailed design, and presentation. The teaching content, methodologies, task requirements, and evaluation criteria remained consistent across groups, with weight configuration serving as the sole experimental variable.

A strict control framework was established to ensure rigor^31^. The same instructional team (one primary instructor and one teaching assistant) taught all three groups, ensuring uniform teaching quality. All three groups used identical assessment indicators, scoring rubrics, and evaluation instruments, differing only in weight configuration^32^. A double-blind design was adopted: students were unaware of their group’s weight configuration and the experimental objectives, preventing expectation effects; evaluators likewise had no knowledge of group assignments and identified student work through a coding system. Five experts who had not participated in instruction independently evaluated student outcomes, providing external criteria for validity testing^33^.

#### Implementation procedures

Implementation proceeded in three phases. The preparation phase (Week 1) comprised student randomization, baseline pretesting, final calibration of evaluation instruments, and standardized evaluator training. Training covered scoring criteria interpretation, practice scoring exercises, and consistency verification^34^. During the implementation phase (Weeks 2–15), all three groups completed coursework under identical content and pacing. Formative assessment data were collected in four stages aligned with the design process: topic selection and investigation (Weeks 2–3), concept development (Weeks 4–7), design refinement (Weeks 8–11), and detailed design (Weeks 12–15). Each stage documented the competency performance most closely matched to that phase^35^, and stage-specific evaluations at the conclusion of each stage tracked students’ competency development trajectories^36^. The evaluation phase (Week 16 onward) comprised the summative assessment. The evaluation panel comprised three university faculty members (associate professor rank or above, ≥ 5 years of teaching experience in landscape architecture) and two industry experts (senior credentials, ≥ 10 years of practice), with disciplinary backgrounds spanning landscape planning and design, plant landscape design, and ecological restoration. Panel members independently scored final design proposals, presentation drawings, and oral defenses according to standardized criteria. For each component, the five evaluators’ scores were averaged to obtain the component score; the three component scores were then weighted (final design proposal 60%, presentation drawings 25%, oral defense 15%) to constitute the summative assessment result. Concurrently, the same five experts conducted independent dimension-by-dimension evaluations of each student’s final outcomes according to the competency framework; the inter-rater reliability coefficient (ICC) was 0.867, confirming scoring reliability.

### Evaluation tool development

#### Competency framework construction

A five-dimensional competency framework for the course was developed through systematic literature review and expert consensus procedures. The framework emerged from a three-round Delphi survey involving 15 experts (8 university faculty, 5 industry professionals, and 2 educational assessment specialists) and achieved high consensus (Kendall’s W = 0.83, *p* < 0.01) on the competency structure presented in Table 1^37^.

**Table 1:** Five-dimensional competency framework for the landscape architecture planning and design course.

Competency dimension	Core definition	Primary indicators	Assessment criteria
Site analysis capability	Comprehensive site assessment and problem identification proficiency	Site investigation, data interpretation, problem identification, opportunity recognition	Analysis comprehensiveness, problem identification accuracy, analysis-design integration
Design thinking and innovation capability	Creative conceptualization and design problem-solving ability	Concept generation, creative development, design innovation, solution formulation	Conceptual openness, idea originality, proposal creativity
Spatial composition capability	Spatial organization and environmental design proficiency	Functional programming, spatial sequencing, environmental integration, scale coordination	Spatial logic, functional efficiency, environmental coherence
Technical application proficiency	Professional knowledge integration and implementation capability	Material specification, construction detailing, topographic manipulation, ecological integration	Technical appropriateness, application logic, implementation viability
Communication and presentation skills	Design communication and professional presentation capability	Graphic representation, model construction, written documentation, oral presentation	Representational clarity, visual effectiveness, communication impact

#### Assessment instrument development and scoring criteria

Based on the five-dimensional competency framework, the research team developed a complete assessment instrument system comprising formative assessment tools, summative assessment tools, and expert evaluation tools. The formative assessment tools consist of four components: design journal evaluation forms, milestone achievement evaluation forms, classroom participation record forms, and design iteration portfolio evaluation forms^38^; these tools track the evolution of students’ thinking, problem-solving strategies, and design iterations across design stages. The summative assessment tools comprise three components: final design proposal evaluation forms, presentation drawing evaluation forms, and oral defense evaluation forms^38^, emphasizing the completeness, professionalism, and innovativeness of design outcomes. The expert evaluation tools required five experts to independently score each of the five competency dimensions of students’ final outcomes according to the same five-dimensional competency framework; the mean score for each dimension served as the expert criterion score for that dimension, and the equally weighted average across the five dimensions constituted the expert overall rating.

Scoring criteria for all the above instruments were developed using a scoring rubrics model, with five performance levels (A–E, corresponding to 90–100 through below 60 points) described for each competency dimension. Each level was accompanied by explicit qualitative descriptors to minimize subjectivity and arbitrariness in scoring; the complete rubric specifications are provided in Supplementary Table S1.

Comprehensive instrument testing was conducted to verify reliability and validity. Content validity was verified through two rounds of expert panel review and revision; the content validity ratios (CVRs) for all five competency dimensions exceeded 0.75. Pilot testing prior to the formal experiment confirmed that internal consistency reliability and inter-rater reliability both met recommended thresholds; specific values are reported in Section “Data quality and baseline characteristics”^39,40^.

### Data collection and analysis methods

#### Data collection procedures

Data collection spanned the entire experiment and encompassed four categories: baseline test data collected prior to the experiment for verifying group equivalence, formative assessment data collected continuously across the four design stages, summative assessment data collected at course completion, and expert evaluation data from five independent experts who scored each student’s final outcomes by dimension according to the competency framework (serving as external criteria for validity analysis^33^). All scores were recorded on a 100-point scale, with weighted totals calculated according to the predetermined configurations.

#### Validity and discrimination analysis

The core analytical methods are validity analysis and discrimination analysis, used to evaluate assessment quality under different weight configurations. Validity analysis reflects the accuracy of measurement results; discrimination analysis captures the precision with which an assessment differentiates among students at varying competency levels.

Validity analysis used Pearson correlation, Fisher’s Z transformation, and content validity ratio calculations, appropriate for the continuous, normally distributed course and expert scores.

1. Pearson correlation analysis: correlation coefficients between total course scores and expert evaluations were calculated for each weight configuration, with higher coefficient values indicating superior evaluation validity. This approach provides a direct measurement of scoring consistency and facilitates validity comparisons across configurations^41^:
  1 $$\begin{array}{*{20}c} {r_{xy} = \frac{{\mathop \sum \nolimits_{i = 1}^{n} \left( {x_{i} - \overline{x}} \right)\left( {y_{i} - \overline{y}} \right)}}{{\sqrt {\mathop \sum \nolimits_{i = 1}^{n} \left( {x_{i} - \overline{x}} \right)^{{2\mathop \sum \nolimits_{i = 1}^{n} \left( {y_{i} - \overline{y}} \right)^{2} }} } }}} \\ \end{array}$$
  where $x_{i}$ represents the student course score, $y_{i}$ represents the expert score, $\overline{x}$ and $\overline{y}$ represent the respective means, and $n$ denotes the sample size.
2. Fisher’s Z transformation: this transformation is applied to compare correlation coefficient significance, which is necessary because correlation coefficients do not follow a normal distribution and require transformation for statistical inference^42^:
  2 $$\begin{array}{*{20}c} {Z = \frac{1}{2}\ln \frac{1 + r}{{1 - r}}} \\ \end{array}$$

Significance testing of differences:

3 $$\begin{array}{*{20}c} {Z_{diff} = \frac{{Z_{1} - Z_{2} }}{{\sqrt {\frac{1}{{n_{1} - 3}} + \frac{1}{{n_{2} - 3}}} }}} \\ \end{array}$$

where $Z_{1}$ and $Z_{2}$ represent Fisher-transformed correlation coefficients and where $n_{1}$ and $n_{2}$ denote the corresponding sample sizes. When comparing dependent correlations derived from the same sample, Hotelling’s t-test was used to determine significance.

(3) Content validity ratio (CVR): evaluates assessment content appropriateness^43^:
  4 $$\begin{array}{*{20}c} {CVR = \frac{{n_{e} - \frac{N}{2}}}{\frac{N}{2}}} \\ \end{array}$$
  where $n_{e}$ represents experts rating an item as “essential” and where $N$ denotes the total number of expert participants.

Discrimination analysis employs multiple verification methods, including discrimination indices, point-biserial correlations, and extreme group comparisons. The 27% criterion was used for high–low group division, a proportion empirically demonstrated to optimize discrimination detection while maintaining sample representativeness and statistical power^43^.

1. Discrimination index (D value): this index measures the ability of an assessment to differentiate students across competency levels^44^:
  5 $$\begin{array}{*{20}c} {D = \frac{{M_{H} - M_{L} }}{{S_{T} }}} \\ \end{array}$$
  where $M_{H}$ and $M_{L}$ represent the high-performing (top 27%) and low-performing (bottom 27%) group means, respectively, and where $S_{T}$ denotes the overall standard deviation.
2. Point-biserial correlation: this method examines the relationships between assessment components and total scores^45^:
  6 $$\begin{array}{*{20}c} {r_{pb} = \frac{{M_{p} - M_{q} }}{{\sigma_{t} }}\sqrt {\frac{{n_{p} \cdot n_{q} }}{{N\left( {N - 1} \right)}}} } \\ \end{array}$$
  where $M_{p}$ and $M_{q}$ represent high and low group means, respectively; ${\upsigma}_{t}$ denotes the overall standard deviation; $n_{p}$ and $n_{q}$ represent group sizes; and $N$ indicates the total sample size.
3. Extreme group analysis: a 27% criterion was implemented to compare performance differences between high- and low-performing groups across competency dimensions, with significance determined through independent t tests^46^.

#### Curve fitting and composite objective function

Curve fitting was used to explore the functional relationship between weight configuration—treated as a continuous variable—and evaluation quality indicators. Educational measurement theory suggests that the relationship between validity and weight configuration may follow an inverted-U nonlinear pattern, and that unidirectional increases in weight may yield diminishing marginal returns. On this basis, the study used the group-level validity coefficients and discrimination indices from the three configurations as observation points, fitted quadratic functions to characterize the direction and approximate magnitude of the trends, and integrated the two indicators through a composite objective function. The main steps were as follows:

1. Fitted equation for formative assessment weight ($X$) and validity ($Y_{1}$): quadratic curve fitting was applied. Preliminary analysis indicated that a quadratic function may best describe this relationship:
  7 $$\begin{array}{*{20}c} {Y_{1} \left( {validity} \right) = a_{1} + b_{1} X + c_{1} X^{2} } \\ \end{array}$$
2. Fitted equation for formative assessment weight ($X$) and discriminatory power ($Y_2$:
  8 $$\begin{array}{*{20}c} {Y_{2} \left( {discrimination} \right) = a_{2} + b_{2} X + c_{2} X^{2} } \\ \end{array}$$
3. Composite objective function, jointly considering validity and discriminatory power:
  9 $$\begin{array}{*{20}c} {F = w_{1} \cdot Y_{1} + w_{2} \cdot Y_{2} } \\ \end{array}$$
  where $w_{1}$ and $w_{2}$ are the weight coefficients for validity and discriminatory power, respectively ($w_{1} + w_{2} = 1$). Following the general principle that validity takes precedence over discriminatory power^47^, this study set $w_{1}$ = 0.6 and $w_{2}$ = 0.4. Because this ratio involves subjective judgment, sensitivity analysis was conducted to examine the response of the optimal configuration as $w_{1}$ varied from 0.4 to 0.8, thereby assessing the robustness of the conclusions.
4. Solving for the optimal weight: through differentiation, setting $\frac{dF}{{dX}} = 0$ and solving for the value of $X$ that maximizes $F$.
5. Sensitivity analysis: because the weight coefficients (${w}_{1}=0.6$, ${w}_{2}=0.4$) involve subjective judgment, the study examined the response of the optimal configuration as ${w}_{1}$ varied from 0.4 to 0.8. Since curve fitting does not provide statistical hypothesis testing, sensitivity analysis served as the primary means of assessing the robustness of the conclusions^48^.

All statistical analyses were performed using SPSS 26.0 (IBM Corp., Armonk, NY, USA) and R 4.0.3 (R Foundation for Statistical Computing, Vienna, Austria; https://www.r-project.org/). All figures were generated by the authors: statistical plots were created using the ggplot2 package (version 3.5.0) in R, and the research methodology framework was created using draw.io (version 26.0.9; https://www.drawio.com/).

## Results and analysis

### Data quality and baseline characteristics

Comprehensive data quality assessments were conducted prior to analysis; baseline characteristics of the three groups are presented in Table 2. Statistical testing confirmed no significant intergroup differences in gender distribution (X^2^ = 0.278, *p* = 0.870), age (F = 0.042, *p* = 0.959), or professional baseline scores (*p* > 0.05), establishing group equivalence at baseline.

**Table 2:** Baseline characteristics across experimental groups.

	Group A (traditional)	Group B (balanced)	Group C (process-oriented)	Statistical significance
Sample size (male/female)	30 (12/18)	30 (11/19)	30 (13/17)	X^2^ = 0.278, p = 0.870
Mean age (years)	20.37 ± 0.89	20.43 ± 0.94	20.40 ± 0.81	F = 0.042, p = 0.959
Drawing proficiency (100-point scale)	78.43 ± 8.67	79.10 ± 8.21	78.77 ± 8.45	F = 0.051, p = 0.950
Spatial cognitive ability (100-point scale)	81.23 ± 7.89	80.97 ± 8.05	81.57 ± 7.92	F = 0.047, p = 0.954
Professional foundation (100-point scale)	82.67 ± 6.98	83.20 ± 7.12	82.33 ± 7.05	F = 0.123, p = 0.885

Cronbach’s α was 0.867 for the formative assessment tools and 0.883 for the summative assessment instruments, both exceeding the recommended threshold of 0.80 and indicating high internal consistency. Intraclass correlation coefficients (ICCs) were 0.815 for formative assessment, 0.842 for summative assessment, and 0.867 for expert evaluation, all exceeding 0.80 and confirming scoring consistency.

**Fig. 2:**
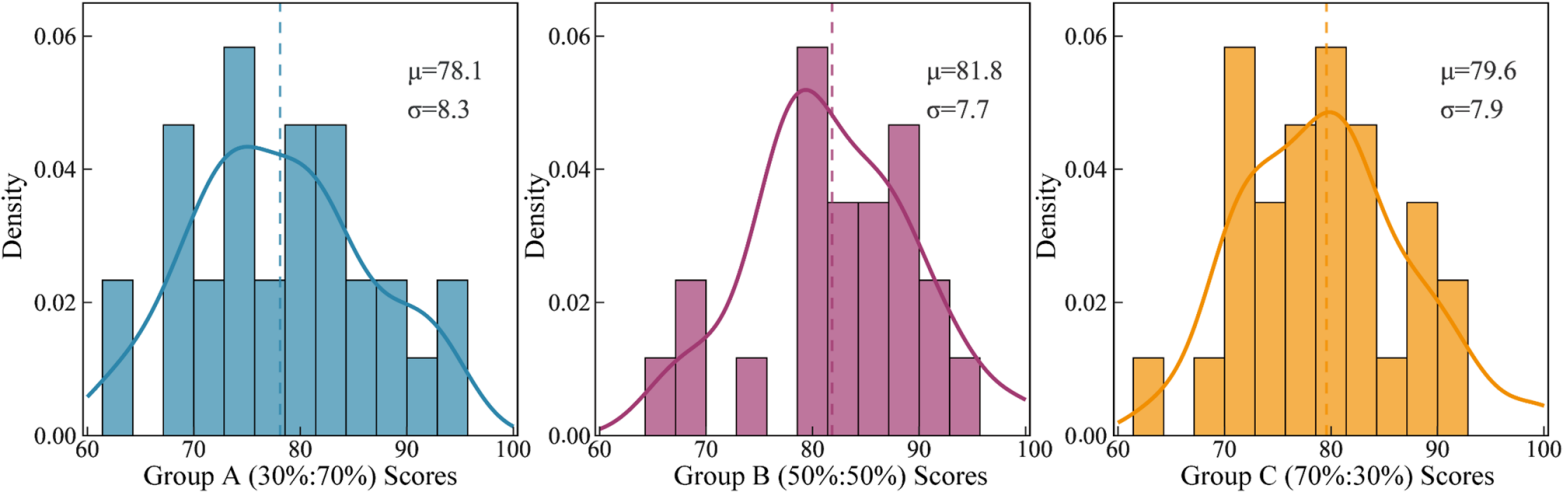
Student score distributions and statistical characteristics under different weight configurations.

Score distributions across the three groups (Fig. 2) revealed distinct patterns under different weight configurations. Group A (traditional) showed concentrated distributions with a high frequency of elevated scores; Group B (balanced) showed relatively uniform distributions approximating normality; and Group C (process-oriented) showed more dispersed distributions, consistent with enhanced discrimination. These distributional differences foreshadowed the discrimination results presented below.

### Weight configuration effects on evaluation validity

**Fig. 3:**
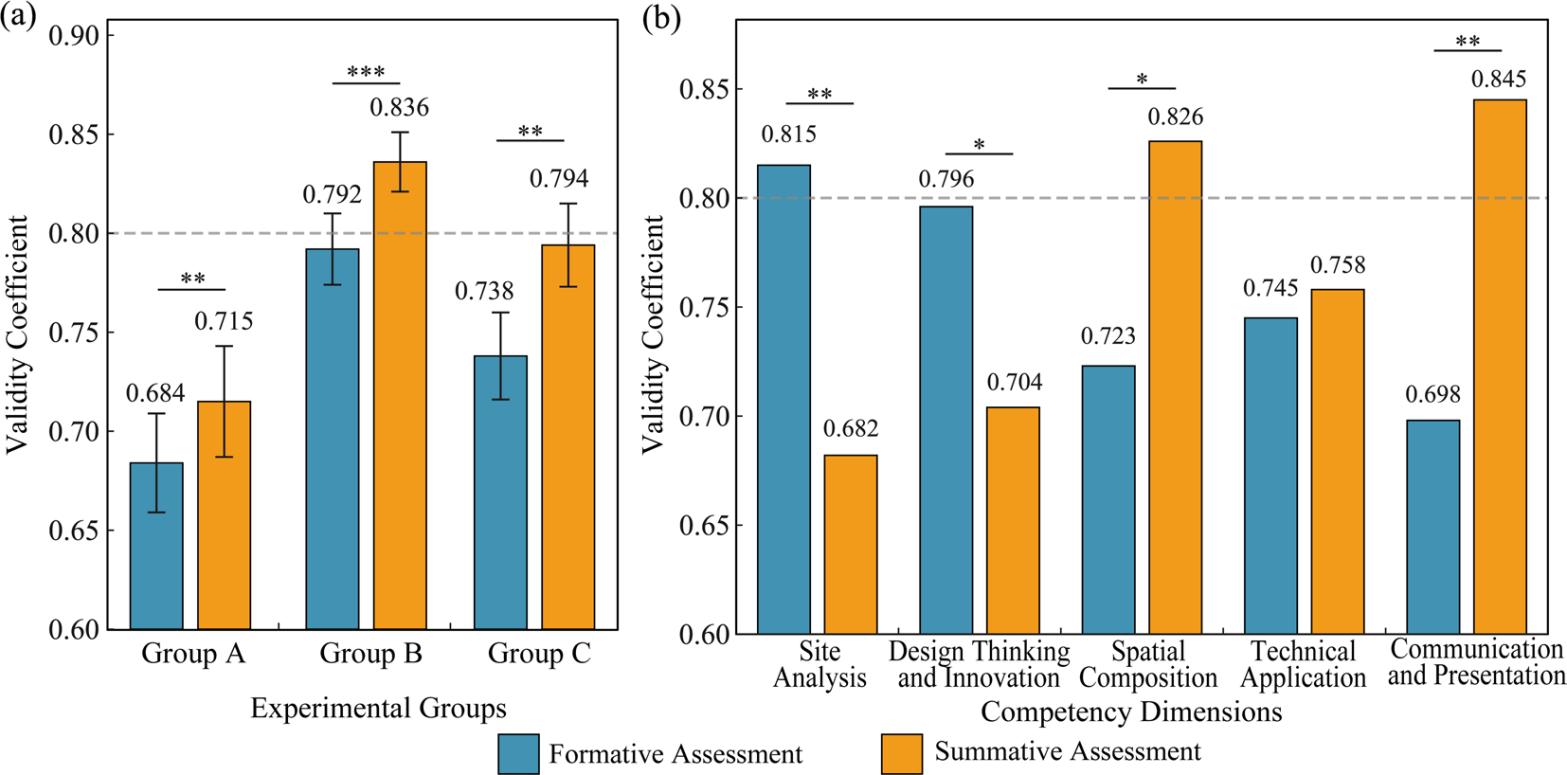
Validity analysis of formative and summative assessment: (**a**) overall validity comparison across the three groups; (**b**) validity differences by competency dimension. *p < 0.05; **p < 0.01; ***p < 0.001.

To examine how weight configurations influence evaluation validity, expert ratings served as external criteria for analyzing the consistency between course scores and expert scores under the three schemes. Pearson correlation analysis indicated that course scores under all three configurations were significantly and positively correlated with expert scores (*p* < 0.001), but the magnitude of the correlations differed substantially. The balanced configuration group (Group B) yielded the highest validity coefficient (r = 0.845), followed by the process-oriented group (Group C, r = 0.776), with the traditional configuration group (Group A) lowest (r = 0.723). The validity difference between Group B and Group A was 0.122, indicating a clear advantage for the balanced configuration. Further analysis of the correlations between each assessment component and expert scores provided additional detail (Fig. 3). Across all groups, formative assessment–expert correlations ranged from 0.684–0.792, whereas summative assessment–expert correlations spanned 0.715–0.836, indicating that both evaluation approaches possess inherent validity. Group B’s formative assessment (r = 0.792) and summative assessment (r = 0.836) both achieved the highest validity levels. This suggests that balanced configurations enhance not only overall validity but also the effectiveness of each assessment component, possibly through complementary interactions between the two modes.

To examine validity differences across competency dimensions, correlations were computed between each dimension’s formative (and separately, summative) assessment score and the corresponding expert score, based on all 90 students; results are presented in Table 3. Site analysis and design thinking showed higher validity under formative than summative assessment, with differences reaching significance at *p* = 0.008 and *p* = 0.021, respectively. This indicates that competencies oriented toward thinking processes are better assessed through formative approaches. Conversely, spatial composition and communication skills exhibited higher validity under summative assessment, reflecting that outcome-oriented competencies are better evaluated through final products. Technical application proficiency showed no significant validity difference between the two modes (*p* = 0.462), suggesting that this dimension manifests equally in both the design process and the final product.

**Table 3:** Validity comparison across competency dimensions by assessment type.

Competency dimension	Formative validity	Summative validity	Significance (*p* value)
Site analysis capability	0.815	0.682	0.008
Design thinking and innovation capability	0.796	0.704	0.021
Spatial composition capability	0.723	0.826	0.012
Technical application proficiency	0.745	0.758	0.462
Communication and presentation skills	0.698	0.845	0.003

To explore the trend between formative assessment weight and validity, the study fitted a quadratic curve using the validity coefficients of the three groups as observation points, with the formative weight percentage ($X$) as the independent variable, yielding the fitted equation: $Y\left(validity\right)=0.182+0.0252X-0.000239{X}^{2}$. The curve passes through all three observation points (Fig. 8a) and indicates that validity first rises then declines as the formative weight increases, approaching a peak near approximately 53%. This trend is consistent with Group B (50%:50%) achieving the highest validity, and further suggests that validity tends to decline once the formative weight exceeds approximately 50%.

**Fig. 4:**
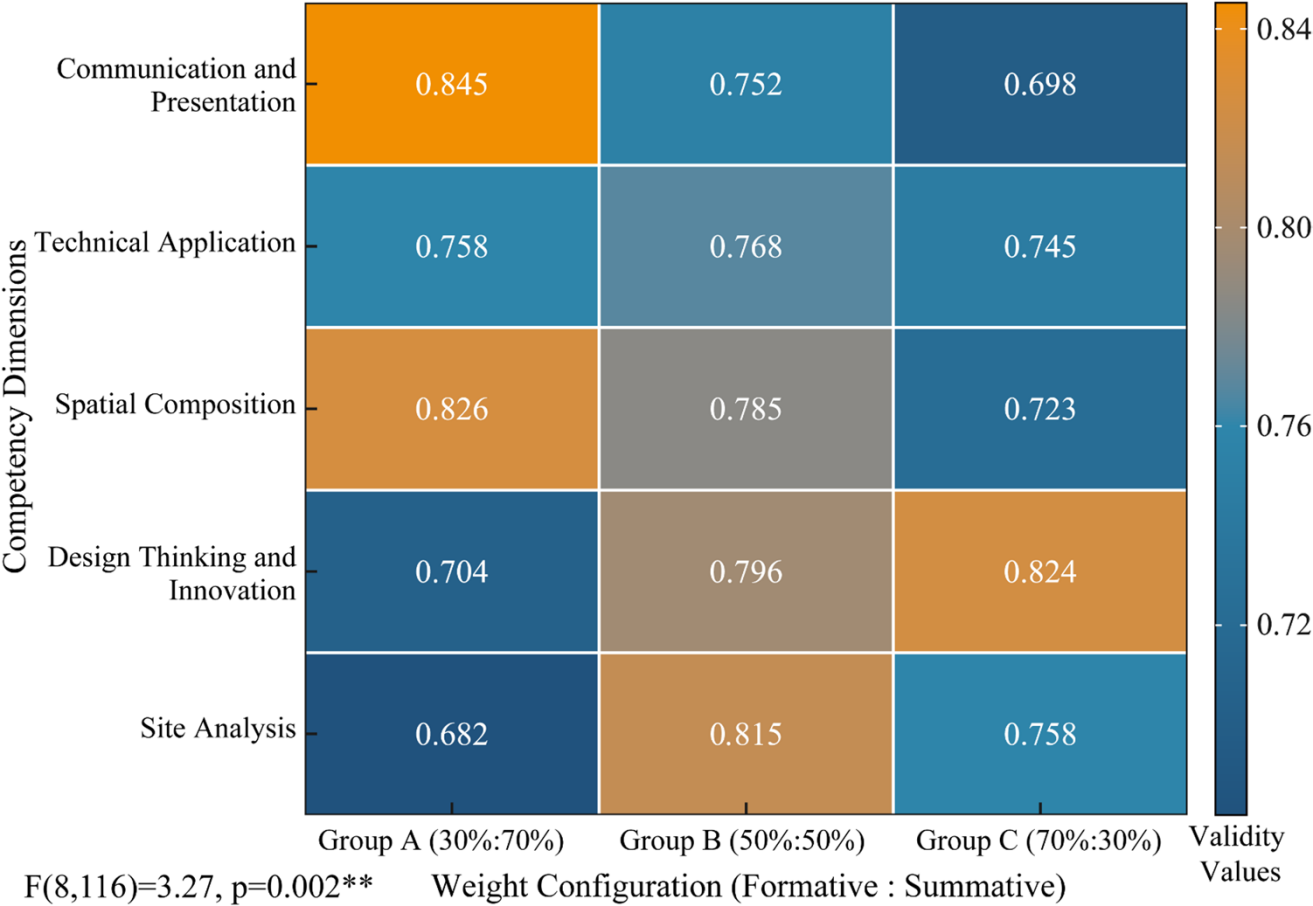
Heatmap of the interactive effects between the weight configuration and evaluation validity by competency dimension.

Weight configuration and competency dimension interact in their effects on evaluation validity, as illustrated in Fig. 4, which reveals that different competencies vary in their sensitivity to weight changes. Site analysis and design thinking showed marked validity gains with increased formative weights, whereas spatial composition and communication showed less sensitivity to weight changes, with occasional slight validity decreases under heavy formative weighting. This interaction pattern supports the case for differentiated evaluation strategies and offers insight into the mechanisms by which weight configuration affects validity.

### Weight configuration effects on competency discrimination

Discrimination indices (D values) were calculated for the three configurations, supplemented by extreme group difference testing. Results are presented in Table 4. The process-oriented configuration group (Group C) achieved the highest discrimination index (D = 0.435), followed closely by the balanced configuration group (Group B) (D = 0.412), with the traditional configuration group (Group A) demonstrating the lowest discrimination (D = 0.325). The t test results for extreme groups indicated that all three configurations achieved highly significant discrimination (*p* < 0.001), with Groups C and B showing elevated t values, reflecting superior discriminatory effectiveness. These findings indicate that increasing the formative proportion enhances discriminatory power, though with diminishing returns. Although Group C achieved the highest discrimination, its advantage over Group B was modest (0.023), whereas both substantially outperformed Group A—a pattern consistent with diminishing marginal returns.

**Table 4:** Discrimination index comparison across weight configurations.

Group	Weight configuration	Discrimination index (D-value)	Extreme group t test
Group A	Formative 30% : Summative 70%	0.325	t = 8.76, p < 0.001
Group B	Formative 50% : Summative 50%	0.412	t = 10.25, p < 0.001
Group C	Formative 70% : Summative 30%	0.435	t = 11.08, p < 0.001

**Fig. 5:**
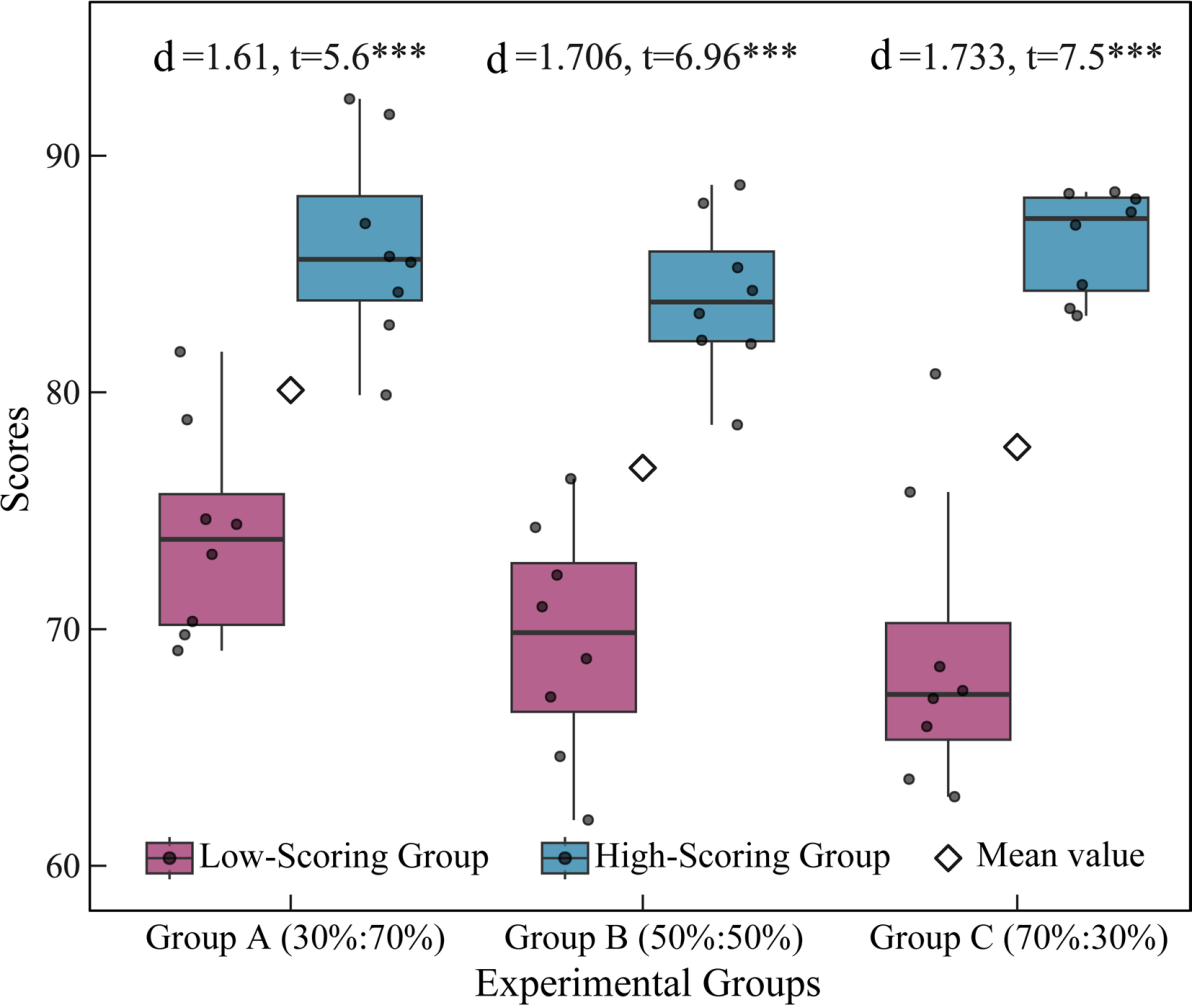
Discrimination effects and statistical test results for high-low score groups under three weight configurations. ***p < 0.001.

Extreme group analysis using the 27% criterion (Fig. 5) further visualized these discrimination effects. Both Group B (86.1 vs. 70.2) and Group C (84.8 vs. 69.5) showed substantially larger score spreads than Group A (85.2 vs. 72.6), with Groups B and C showing comparable effects. This analysis corroborates the finding that increased formative proportions improve discriminatory power.

**Fig. 6:**
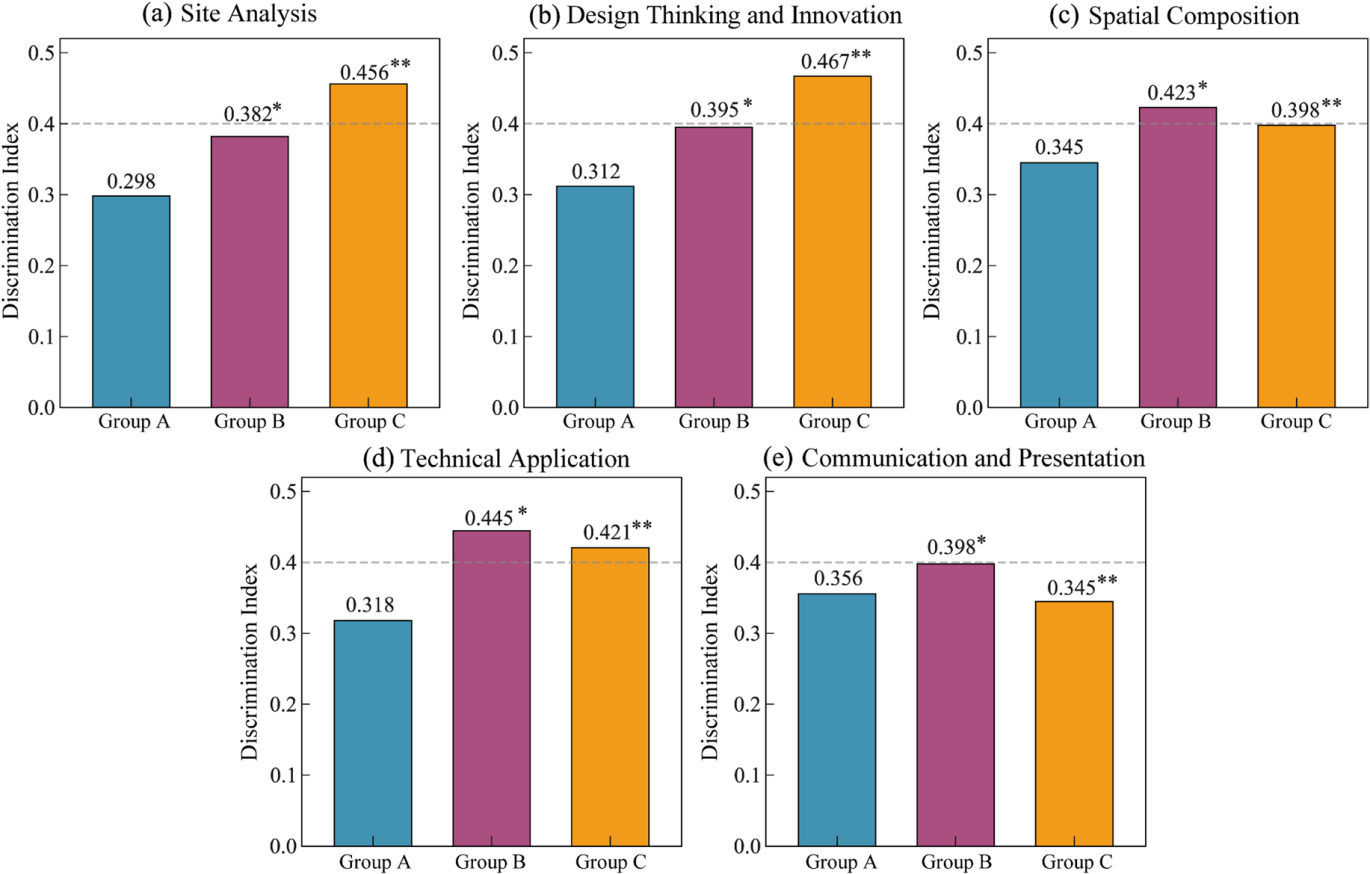
Discrimination indices of the five competency dimensions under different weights. *p < 0.05; **p < 0.01.

Discrimination indices across competency dimensions also varied under different configurations, as illustrated in Fig. 6. Site analysis and design thinking showed marked gains in discrimination with increased formative proportions, with Group C (70%:30%) significantly outperforming Group A (30%:70%). This indicates that discrimination for process-oriented competencies depends more heavily on formative assessment. Spatial composition and communication showed smaller discrimination variations across configurations, with Group B (50%:50%) achieving optimal balance. Technical application proficiency reached peak discrimination under the balanced configuration (Group B), suggesting that different competency dimensions have distinct discriminatory profiles that cannot be fully captured by a single assessment mode.

The same curve fitting approach was applied to the relationship between formative assessment weight and discriminatory power, yielding the fitted equation: $Y\left(discrimination\right)=0.075+0.0108X-0.00008{X}^{2}$. The fitted trend (Fig. 8b) shows that discriminatory power continues to rise as the formative assessment weight increases, but the rate of increase gradually decelerates, approaching a plateau near a weight of approximately 67%. This direction is consistent with the experimental observation that Group C (70%:30%) achieved the highest discriminatory power, indicating that from the perspective of discriminatory power, a higher formative assessment proportion is advantageous, although the marginal benefit exhibits diminishing returns.

### Assessment structure and comprehensive weight configuration analysis

Formative and summative assessments showed a moderate positive correlation (r = 0.683, *p* < 0.01), indicating partial overlap alongside unique contributions from each mode. Correlation patterns across competency dimensions are detailed in Table 5. Spatial composition (r = 0.725) and technical application (r = 0.694) showed higher correlations between the two assessment modes, whereas site analysis (r = 0.592) and communication (r = 0.583) showed lower correlations.

**Table 5:** Formative-summative assessment correlations by competency dimension.

Competency dimension	Correlation coefficient (r)	Significance (*p* value)
Site analysis capability	0.592***	< 0.001
Design thinking and innovation capability	0.637***	< 0.001
Spatial composition capability	0.725***	< 0.001
Technical application proficiency	0.694***	< 0.001
Communication and presentation skills	0.583***	< 0.001
Overall assessment	0.683***	< 0.001

***p < *0.001.*

**Fig. 7:**
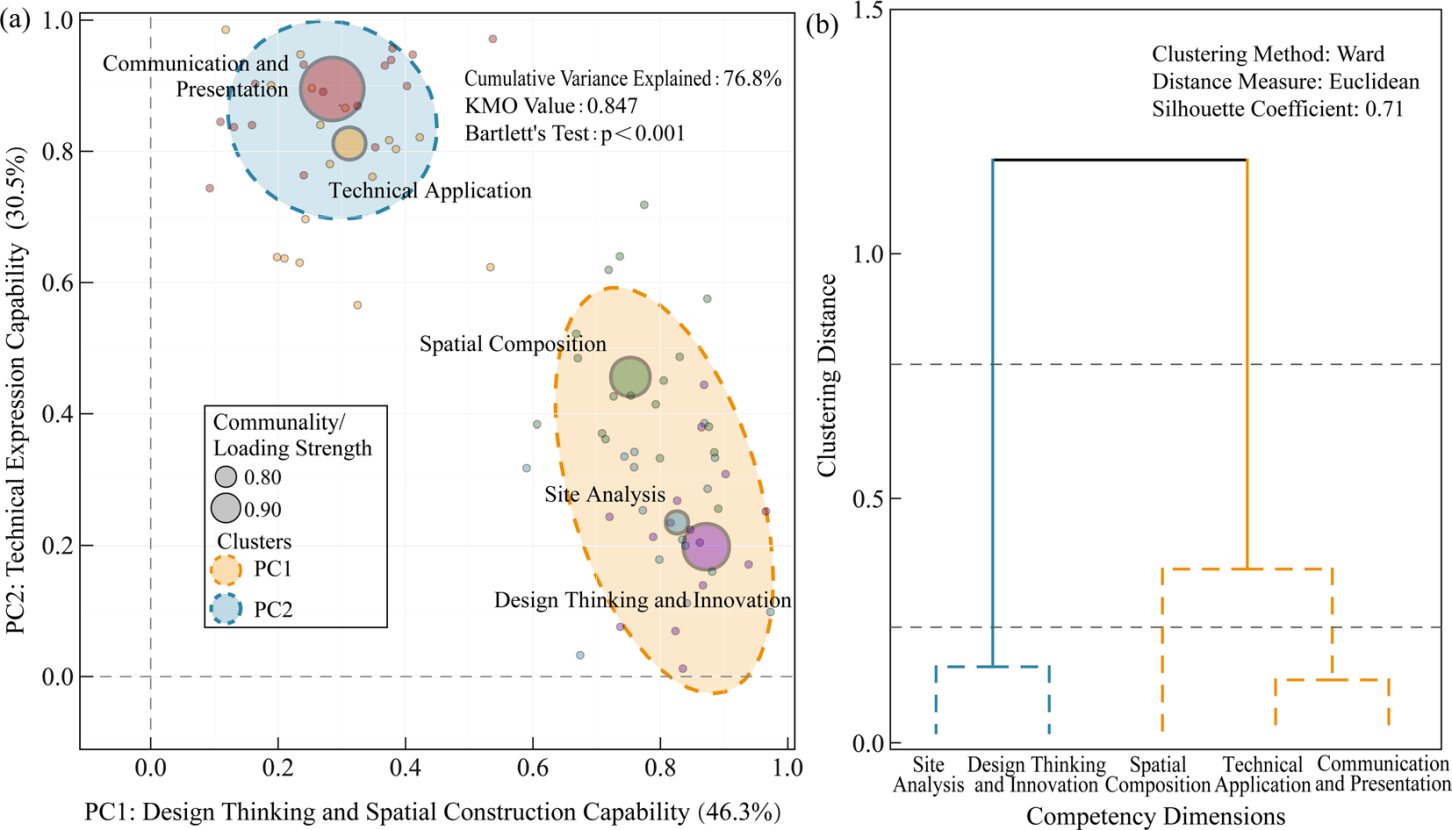
Five-dimensional competency structure analysis: (**a**) factor analysis scatter plot with principal component loadings; (**b**) hierarchical clustering dendrogram.

Factor analysis of the underlying competency structure revealed two principal components explaining 76.8% of total variance (Fig. 7). After orthogonal rotation, the first component comprised site analysis (0.826), design thinking (0.872), and spatial composition (0.753), interpretable as “design thinking and spatial construction capability” and accounting for 46.3% of variance. The second component consisted of technical application (0.812) and communication (0.895), interpretable as “technical expression capability,” explaining 30.5% of variance. This structure suggests that the five dimensions can be condensed into two core competency clusters, providing a basis for streamlined evaluation frameworks.

Substituting the fitted equations from Sections “Weight configuration effects on evaluation validity” and “Weight configuration effects on competency discrimination” into the composite objective function (Eq. 9, with ${w}_{1}=0.6$, ${w}_{2}=0.4$), the expanded expression is:

$$\begin{aligned} F = & 0.6(0.182 + 0.0252X - 0.000239X^{2} ) + 0.4(0.075 + 0.0108X - 0.00008X^{2} ) \\ = & 0.139 + 0.01944X - 0.000175X^{2} \\ \end{aligned}$$

Taking the derivative:

$$\frac{dF}{dX}=0.0194-0.000350X$$

**Fig. 8:**
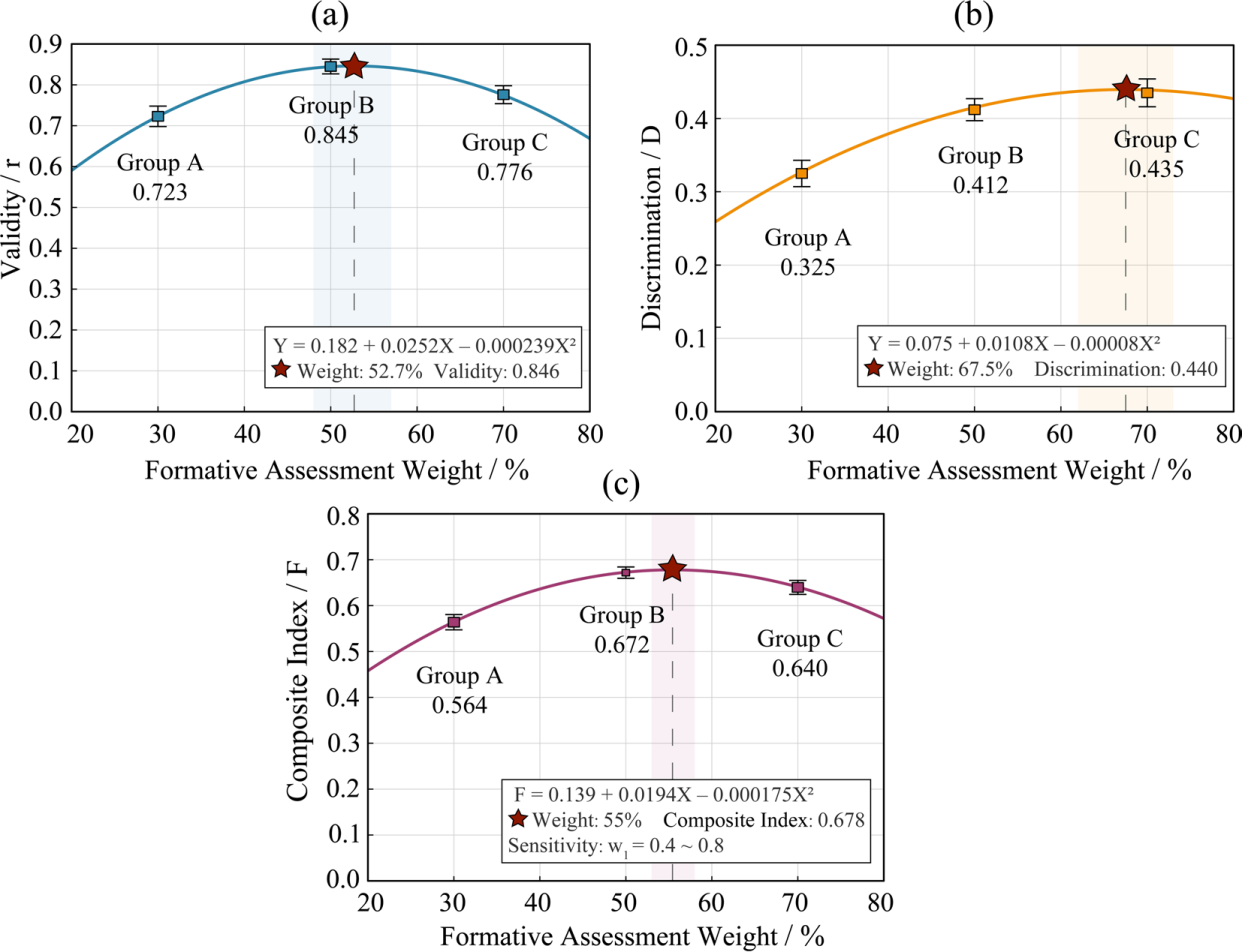
Nonlinear relationships between formative assessment weights and validity-discrimination performance and optimal weight determination.

Setting $\frac{dF}{dX}=0$ yields X ≈ 55.4. When validity and discriminatory power are jointly considered, the composite indicator approaches its optimum at a formative assessment weight of approximately 55% (Fig. 8c). The direction of this optimum depends on the setting of ${w}_{1}$ and ${w}_{2}$. To examine the sensitivity of the conclusion to this parameter, the study systematically varied ${w}_{1}$ from 0.4 to 0.8. As ${w}_{1}$ moved from 0.4 to 0.8, the optimal formative assessment weight shifted from approximately 58% down to approximately 54%, consistently falling within a roughly balanced, slightly process-leaning interval—indicating that the conclusion is robust to the parameter setting. The trend curves are uniquely determined by three observation points and therefore constitute descriptive fitting. The robustness of the conclusion rests primarily on the sensitivity analysis: the stability of the optimal weight under parameter variation is more persuasive than the fitted curve itself. Under the present experimental conditions, a formative-to-summative configuration of approximately 55:45 demonstrated stronger overall performance. This direction was derived from data on a specific course and student population; its applicability in other educational contexts warrants further investigation. For practical purposes, a 50:50 balanced configuration is also a viable option.

## Discussion and implications

### Nonlinear effects of weight configuration on assessment quality

As shown in Section “Weight configuration effects on evaluation validity”, the balanced configuration achieved the highest validity, indicating that incorporating information from the two assessment modes in roughly equal proportions yields the highest consistency with independent expert evaluations. This section explores possible mechanisms from a theoretical perspective.

This result can be understood first through the information complementarity of the two assessment modes. Formative assessment excels at capturing the formation and evolution of design reasoning, whereas summative assessment excels at judging the overall quality of the final product^23^. A balanced configuration provides adequate space for both types of information in the composite score; when either component is weighted too lightly, the corresponding information is diluted and construct validity suffers. A complementary explanation involves bias cancellation. In design education, summative assessment may overestimate students who excel in presentation but lack depth of thinking, because the visual impact of the final product can mask weaknesses in preliminary analysis. Formative assessment, conversely, may overestimate students who are highly engaged in the process yet produce mediocre final outcomes, because active process performance does not necessarily translate into high-quality design products. If these opposing biases exist, a balanced configuration may allow them to cancel out, bringing the composite score closer to students’ true competency levels. Related research in medical education has reported similar findings: formative and summative assessment each have distinct advantages in predicting overall competency, and using either alone is less effective than combining them^49^.

Regarding discriminatory power, the process-oriented configuration (70%:30%) achieved the highest value (D = 0.435), but the gap with the balanced configuration (D = 0.412) was small; both substantially outperformed the traditional configuration (D = 0.325) (Table 4). The underlying mechanism is that formative assessment, through multi-timepoint, multi-dimensional data collection, more precisely reveals differences in students’ thinking strategies and problem-solving pathways^50^; these differences tend to be compressed in the single cross-section of summative assessment. However, when the formative proportion increased from 50 to 70%, the gain in discriminatory power was modest, exhibiting diminishing marginal returns. This diminishing effect is related to the inherent subjectivity and context-dependence of formative assessment. As Yang^51^ has noted, evaluators’ judgments of students’ process performance are influenced by factors such as observational perspective and interaction frequency. As the formative proportion increases, the cumulative effect of these error sources in the total score grows, progressively offsetting the marginal gains in discriminatory power.

The response patterns of validity and discriminatory power to weight configuration are not entirely congruent. Validity approached its optimum near a formative assessment weight of approximately 53%, while discriminatory power continued to rise slowly at higher proportions (with a fitted trend curve peak at 67%). After integrating the two indicators through the composite objective function, a formative weight of approximately 55% emerged as the direction of stronger overall performance; sensitivity analysis confirmed that this direction remained stable across different priority settings for validity and discriminatory power. This finding aligns with Brown’s observation that validity and discriminatory power exist in a negotiable, balanceable relationship [50]. For practice, these results suggest that assessment reform in the “Landscape Architecture Planning and Design” course may take a roughly balanced, slightly process-leaning configuration as its starting point. The specific proportions may vary with course conditions—for example, courses emphasizing preliminary research and conceptual reasoning may suit a higher formative proportion, while courses emphasizing construction documentation and technical implementation may call for a more balanced configuration.

### Differential matching mechanisms between competency dimensions and assessment modes

The empirical data (Table 3) show that competency dimensions exhibited systematic, differential responses to the two assessment modes: site analysis and design thinking showed higher validity under formative assessment, spatial composition and communication showed higher validity under summative assessment, and technical application proficiency showed no significant difference between the two modes. These data point to a matching pattern: competency dimensions oriented toward cognitive processes are better captured by formative assessment, while those oriented toward integrative outcomes are better captured by summative assessment.

Design cognition theory and reflective practice theory provide a framework for understanding this pattern. Cross argued that designers’ ways of knowing differ from scientific reasoning and humanistic interpretation, their defining characteristic being the knowledge generated through the act of making^24^; Schön further distinguished between reflection-in-action and reflection-on-action in professional practice^26^. Site analysis confronts a quintessentially ill-structured problem^25^: students collect and screen multi-source information over an extended period, establish relational links among site elements, and distill design opportunities from existing conditions. The quality of problem framing in this process is largely tacit and virtually impossible to infer retroactively from a single site analysis drawing. Design thinking exhibits a similar pattern: concept generation hinges on repeated alternation between divergent and convergent thinking—a cognitive iteration that constitutes the core of this capability yet is compressed in the final scheme into what appears to be a determinate result. Both competency types depend heavily on what Schön termed reflection-in-action—designers continuously revise their understanding and reconstruct problem frames during investigation and concept development. These reflective trajectories constitute a core dimension of professional competency in design, and formative assessment achieves higher validity on these dimensions precisely because it documents this process of “thinking through doing” (site analysis: r = 0.815 vs. 0.682, *p* = 0.008; design thinking: r = 0.796 vs. 0.704, *p* = 0.021). Research on tacit knowledge in design education corroborates this point: many critical cognitive activities in the design process carry an ineffable, tacit quality that can only be effectively captured through process-oriented observation and dialogue^52^. By contrast, spatial composition and communication skills are more fully expressed as integrative products of completed action. A spatial scheme requires integrating functional layout, circulation, spatial sequence, and other elements into a coherent whole whose quality can only be fully judged in the finished state; drawing presentation and oral defense are performance tasks for which evaluators must observe the actual behavior before rendering a valid judgment. The holistic, outcome-oriented character of summative assessment is well suited to these competency types.

Technical application proficiency showed no significant validity difference between the two assessment modes (*p* = 0.462), a result that, far from contradicting the theoretical framework above, provides a valuable calibration point. Technical application involves both decision-making during the design process (such as material selection and construction detailing, within the domain of reflection-in-action) and demonstration of implementation effects in the final scheme (within the domain of post-action product); it spans process and outcome, and each assessment mode captures a different facet. This also explains why the discrimination index for technical application reached its highest level under the balanced configuration—only by incorporating both types of assessment information can the full competency profile on this dimension be revealed.

These matching patterns suggest that, beyond determining the overall weight configuration, assessment schemes for design courses should consider the compatibility between individual competency dimensions and assessment modes, to avoid weights that are reasonable at the aggregate level but produce information masking at the dimension level.

### Contributions and limitations

The principal contribution of this study is an analytical pathway bridging educational measurement frameworks with design education practice: using independent expert ratings by dimension as external criteria, decomposing assessment quality into validity and discriminatory power, and on that basis revealing how individual competency dimensions respond differentially to assessment modes along with the cognitive roots of those responses. This pathway can be adapted by related disciplines such as architectural design, urban design, or product design, although differences in competency structure across disciplines may shift the direction of the optimal weight configuration.

Several limitations warrant acknowledgment. At the sample level, the study was conducted at a single provincial university, which limits generalizability. As noted in the Introduction, design education assessment is profoundly shaped by institutional traditions and studio culture^17,18^, and the optimal weight configuration may differ across educational systems: institutions with deep-rooted studio teaching traditions and well-established formative feedback mechanisms may have a higher baseline validity for formative assessment, shifting the starting point of a balanced configuration accordingly, whereas for Chinese universities currently undergoing assessment-model transitions the directional reference offered by this study has more immediate practical value. Future multi-center and cross-cultural comparative research will help test the robustness of this effect. At the design level, the curve fitting was based on three observation points; adding intermediate configurations would yield a finer-grained response curve. The observation period spanned one semester; longitudinal tracking would help examine the long-term effects of weight configuration on competency development. In addition, incorporating qualitative data—such as student interviews and instructor reflections—would illuminate the mechanisms through which weight configuration shapes assessment quality.

## Conclusion

Through a randomized controlled experiment in an authentic educational setting, this study compared the effects of three formative–summative weight configurations on assessment quality in the “Landscape Architecture Planning and Design” course. The results revealed nonlinear relationships between weight configuration and assessment quality, and identified systematic differences in how individual competency dimensions respond to the two assessment modes.

1. Weight configuration exerts a significant influence on evaluation validity, displaying an inverted-U nonlinear pattern. The balanced configuration (50%:50%) yielded higher evaluation validity (r = 0.845) than the traditional configuration (30%:70%, r = 0.723) and the process-oriented configuration (70%:30%, r = 0.776). Curve fitting indicated that validity approached its optimum near a formative weight of approximately 53%; both excessively high and low formative weights reduced validity.
2. The effect of weight configuration on discriminatory power exhibits diminishing marginal returns. The process-oriented configuration achieved the highest discriminatory power (D = 0.435), followed by the balanced configuration (D = 0.412), with the traditional configuration lowest (D = 0.325). Increasing the formative proportion strengthens discriminatory power, but the rate of improvement decelerates once the proportion exceeds 50%. When validity and discriminatory power are considered jointly, a formative weight of approximately 55% demonstrated stronger overall performance; sensitivity analysis confirmed that this finding is robust to parameter settings.
3. Different competency dimensions exhibit systematic matching differences with assessment modes. Site analysis and design thinking showed higher validity under formative assessment, spatial composition and communication performed better under summative assessment, and technical application proficiency performed similarly under both modes. Factor analysis further indicated that the five dimensions can be condensed into two principal components: “design thinking and spatial construction capability” and “technical expression capability.”

On the basis of these findings, we recommend that design courses, when refining their assessment schemes, take a roughly balanced, slightly process-leaning weight configuration as a starting point, carry out context-specific adaptation in light of each course’s competency structure and pedagogical traditions, and use validity and discriminatory power as ongoing calibration indicators of assessment quality. This study offers an experimentally grounded alternative to the long-standing practice of configuring weights on the basis of experiential intuition. The matching patterns between competency dimensions and assessment modes revealed here also provide educators with a theoretical reference for designing differentiated assessment schemes tailored to the competency structure of their courses.

## Supplementary Information

Below is the link to the electronic supplementary material.

Supplementary Material 1

## Data Availability

The datasets generated and analyzed during the current study are available from the corresponding author upon reasonable request. Due to privacy and ethical restrictions related to student performance data, the datasets cannot be made publicly available.
