# Supplementary material for: Effects of formative-summative assessment weight configurations on evaluation validity and discriminatory power in landscape architecture planning and design courses
Source: Sci Rep. 2026 Apr 27;16:19299. doi: 10.1038/s41598-026-50554-7 (PMC13284382; doi:10.1038/s41598-026-50554-7)
Supplement: Supplementary file 1 — Supplementary Material 1 [file 41598_2026_50554_MOESM1_ESM.docx]

**Supporting information**

**S1 Table. Competency Assessment Rubrics.**

| Competency Dimension | Performance Level | Assessment Criteria | Score Range |
| --- | --- | --- | --- |
| Site Analysis Capability | Excellent (A) | Demonstrates comprehensive and sophisticated site condition analysis with accurate identification of critical issues and discovery of unique opportunities. Exhibits rigorous analytical logic with insightful conclusions. Creatively applies analytical findings to inform design development, generating distinctive conceptual frameworks. | 90-100 |
|  | Good (B) | Provides thorough site condition analysis with identification of primary issues and recognition of general opportunities. Demonstrates clear analytical logic with sound conclusions. Effectively utilizes analytical results to guide design development, forming coherent conceptual frameworks. | 80-89 |
|  | Satisfactory (C) | Conducts fundamental site condition analysis with identification of basic issues. Demonstrates logical analytical processes with acceptable conclusions. Partially integrates analytical results into design development, establishing rudimentary conceptual frameworks. | 70-79 |
|  | Needs Improvement (D) | Provides superficial site analysis with inadequate issue identification. Demonstrates limited analytical logic with simplistic conclusions. Weakly connects analytical findings to design development, producing unclear conceptual frameworks. | 60-69 |
|  | Unsatisfactory (E) | Demonstrates severely inadequate site analysis with failure to identify critical issues. Lacks analytical logic with incorrect or absent conclusions. Cannot effectively translate analysis into design guidance, lacking coherent conceptual frameworks. | <60 |
| Design Thinking and Innovation | Excellent (A) | Generates distinctive and original design concepts demonstrating broad and sophisticated thinking. Produces highly creative proposals with innovative and effective problem-solving approaches. Consistently transcends conventional thinking paradigms, exhibiting exceptional innovation capacity. | 90-100 |
|  | Good (B) | Develops original design concepts with reasonably broad thinking scope. Creates proposals demonstrating meaningful innovation with effective problem-solving strategies. Occasionally transcends conventional approaches, showing solid innovation capacity. | 80-89 |
|  | Satisfactory (C) | Produces acceptable design concepts with adequate thinking scope. Generates conventional proposals with fundamentally viable problem-solving approaches. Shows limited innovation, primarily following established methodologies. | 70-79 |
|  | Needs Improvement (D) | Creates basic design concepts with restricted thinking scope. Produces proposals lacking novelty with elementary problem-solving approaches. Demonstrates minimal innovation, relying entirely on conventional methodologies. | 60-69 |
|  | Unsatisfactory (E) | Fails to articulate coherent design concepts with severely constrained thinking. Produces outdated or imitative proposals unable to address problems effectively. Shows no innovation capacity, exhibiting confused thinking or plagiaristic tendencies. | <60 |
| Spatial Composition Capability | Excellent (A) | Creates sophisticated and innovative spatial arrangements with scientifically organized functional zoning. Develops fluid circulation systems with rich, hierarchical spatial sequences. Achieves comprehensive and distinctive overall spatial organization. | 90-100 |
|  | Good (B) | Develops well-considered spatial arrangements with logically organized functional zoning. Creates efficient circulation systems with varied spatial sequences. Achieves coherent overall spatial organization. | 80-89 |
|  | Satisfactory (C) | Produces fundamentally sound spatial arrangements with adequately organized functional zoning. Establishes functional circulation systems with basic spatial sequence variation. Achieves generally coherent overall spatial organization. | 70-79 |
|  | Needs Improvement (D) | Creates problematic spatial arrangements with inadequately organized functional zoning. Develops inefficient circulation systems with monotonous spatial sequences. Achieves incomplete overall spatial organization. | 60-69 |
|  | Unsatisfactory (E) | Produces chaotic spatial arrangements with incorrectly organized functional zoning. Creates dysfunctional circulation systems with absent spatial sequences. Fails to achieve coherent or balanced overall spatial organization. | <60 |
| Technical Application Proficiency | Excellent (A) | Demonstrates sophisticated and innovative material selection with scientifically sound construction design. Applies advanced ecological technologies effectively. Achieves seamless integration between technical solutions and design concepts, exhibiting exceptional technical competency. | 90-100 |
|  | Good (B) | Shows appropriate material selection with sound construction design principles. Applies ecological technologies reasonably. Achieves good integration between technical solutions and design concepts, demonstrating solid technical competency. | 80-89 |
|  | Satisfactory (C) | Demonstrates adequate material selection with fundamentally sound construction design. Applies basic ecological technologies appropriately. Achieves general integration between technical solutions and design concepts, showing acceptable technical competency. | 70-79 |
|  | Needs Improvement (D) | Shows inadequate material selection with questionable construction design principles. Applies ecological technologies inappropriately. Achieves poor integration between technical solutions and design concepts, demonstrating limited technical competency. | 60-69 |
|  | Unsatisfactory (E) | Demonstrates inappropriate material selection with unscientific construction design. Fails to apply ecological technologies correctly or omits them entirely. Shows disconnection between technical solutions and design concepts, exhibiting minimal technical competency. | <60 |
| Communication and Presentation | Excellent (A) | Produces precise and articulate graphic communication with exceptional visual impact. Delivers comprehensive and compelling design narrative with clear, persuasive presentation logic. Responds expertly to questions and critiques with sophisticated professional discourse. | 90-100 |
|  | Good (B) | Creates clear and accurate graphic communication with strong visual effectiveness. Provides comprehensive design narrative with logical presentation structure. Responds competently to questions and critiques with sound professional reasoning. | 80-89 |
|  | Satisfactory (C) | Develops adequate graphic communication with acceptable visual presentation. Delivers basic design narrative with generally logical presentation structure. Responds appropriately to questions and critiques with fundamental professional understanding. | 70-79 |
|  | Needs Improvement (D) | Produces unclear or imprecise graphic communication with poor visual effectiveness. Provides incomplete design narrative with unclear presentation logic. Struggles to respond effectively to questions and critiques, demonstrating limited professional articulation. | 60-69 |
|  | Unsatisfactory (E) | Creates imprecise and unclear graphic communication with inadequate visual presentation. Delivers insufficient or incorrect design narrative with confused presentation logic. Cannot respond appropriately to questions and critiques, lacking professional communication competency. | <60 |
